# The Comparison of HHHFNC and NCPAP in Extremely Low-Birth-Weight Preterm Infants After Extubation: A Single-Center Randomized Controlled Trial

**DOI:** 10.3389/fped.2020.00250

**Published:** 2020-06-26

**Authors:** Jia Chen, Yingyi Lin, Lanlan Du, Mengmeng Kang, Xiufang Chi, Zhu Wang, Ying Liu, Weiwei Gao, Jie Yang, Yunbin Chen

**Affiliations:** Neonatal Department, Guangdong Women and Children Hospital, Guangzhou, China

**Keywords:** extremely low-birth-weight preterm infants, heated humidified high-flow nasal cannula, nasal continuous positive airway pressure, preterm infant, respiratory distress syndrome

## Abstract

**Objectives:** To compare the clinical efficacy of heated, humidified high-flow nasal cannula (HHHFNC) and nasal continuous positive airway pressure (NCPAP) in extremely low-birth-weight preterm infants (ELBWI) after extubation.

**Methods:** This trial included 94 extremely low-birth-weight infants (ELBWI), within 7 days after birth, and prepared for tracheal extubation and a change to non-invasive ventilation in the neonatal intensive care unit (NICU) admitted to our hospital from January 2015 to December 2018, with 48 infants in the HHHFNC group and 46 infants in the NCPAP group. Reintubation rate within 72 h after initial extubation, total ventilation time, non-invasive ventilation time, total oxygen inhalation time, and the time to reach full enteral feeding were the primary outcome measures. Total intestinal feeding time, average weight gain rate, days of hospitalization, costs of hospitalization, and complication rates, including nasal injury, IVH, BPD, NEC, ROP, and PDA, were used as secondary outcomes. Data were analyzed using Student's *t*-test or the Mann-Whitney *U*-test with a Chi-square test or Fisher's exact test, as appropriate, in SPSS (25.0).

**Results:** HHHFNC not only shortened the oxygen exposure time but also effectively reduced the incidence of nasal injury (6.25 vs. 36.96%) and NEC (10.42 vs. 28.26%) (*P* < 0.05). Additionally, HHHFNC achieved a significant advance in the time to reach full enteral feeding (31.24 ± 11.35 vs. 34.21 ± 14.09 days); increased the average weight gain rate (16.07 ± 3.10 vs. 13.74 ± 4.21) and reduced the days of hospitalization (73.45 ± 18.84 vs. 79.24 ± 19.75), with a lower cost of hospitalization (16.04 ± 3.64 vs.18.79 ± 4.13) thousand dollars (all *P* < 0.05).

**Conclusions:** Compared with NCPAP, HHHFNC was effective in preventing extubation failure in mechanically ventilated preterm ELBWI. HHHFNC shortens oxygen consumption time and significantly reduces the incidence of nasal injury and necrotizing enterocolitis; moreover, it can also reduce the length of stay and the hospitalization costs.

## Introduction

The birth and survival rates of premature infants, especially extremely low-birth-weight infants (ELBWI), have brought about gradual increases in short- and long-term complications. The establishment of good ventilation after birth is the basis for the survival of premature infants, especially for ELBWI. Both the earlier gestational age and the lower birth weight can make it difficult to establish spontaneous breathing and may also increase the incidence of respiratory distress (1).

Invasive mechanical ventilation is widely used in neonatal intensive care units (NICUs). However, long-term invasive mechanical ventilation can lead to ventilator-related lung injuries, including pressure injuries, volume injuries, and ventilator pneumonia. In later stages, it may even lead to severe infection and bronchopulmonary dysplasia (BPD) (2), seriously affecting the long-term quality of life of infants. Therefore, extubation is recommended as soon as possible for neonates, especially premature babies, to avoid the potential damages caused by invasive ventilation as much as possible. However, early extubation is prone to extubation failure, resulting in changes in the condition of the child and more local damage. Non-invasive ventilation after extubation helps prevent possible apnoea, respiratory failure, and re-intubation.

Nasal continuous positive airway pressure (NCPAP), as the current mainstream non-invasive ventilation model, has been widely used in clinical practice to prevent tube failure in preterm infants (3, 4). However, complications (i.e., nasal injury and NEC) caused by NCPAP have a great impact on clinical outcomes (5). Humidified high-flow nasal cannula (HHHFNC) is another globally non-invasive respiratory support model for the prevention of extubation in preterm infants (6), as the use of HHHFNC may be associated with reduced respiratory function, increased ventilation efficiency, and reduced intubation requirements in children with inadequate respiratory function (7).

As primary respiratory support for preterm infants with respiratory distress, HHHFNC and NCPAP are associated with a lower incidence of nasal trauma (8). In this regard, a pilot study suggested that HHHFNC may be as effective as NCPAP in preventing endotracheal ventilation in premature infants in the primary treatment of respiratory distress syndrome (gestational age < 35 weeks and birth weight > 1,000 g) (9). However, there is still a lack of clinical research on the effects of the two non-invasive ventilation modes as the preferred respiratory support model for ELBWI extubation.

This study investigated the clinical efficacy of HHHFNC compared with NCPAP for ELBWI, aiming to explore a more effective mode of non-invasive ventilation for ELBWI.

## Methods

### Ethics Approval

This single-institution prospective randomized clinical trial was conducted in our hospital from January 2015 to December 2018. This study was approved by the Ethics Committee and the institutional review board of the Guangdong Women and Children Hospital (Guangzhou, China). Parental written informed consent was required before delivery of the potentially eligible infants. The authors confirm that all ongoing and related trials for this intervention are registered (ChiCTR1900028092).

### Participants and Design

Considering α = 0.05, power = 80%, an attrition rate of 5% and Cohen's *d* = 0.37 (medium effect size), a 92-subject sample size was determined for the study.

We included infants who met the following criteria in this hospital. The inclusion criteria were as follows: (1) gestational age < 32 weeks, body weight < 1,000 g; (2) the preterm neonates were diagnosed with RDS, supported by invasive ventilation and entered the NICU within 7 days after birth and prepared for tracheal extubation and a change to non-invasive ventilation; and (3) agreement by the family to sign the informed consent form.

The standard of intubation: Infants can be intubated if they have the following conditions: severe apnea (>6 episodes, stimulation within 6 h, or >1 bag and mask ventilation); arterial carbon dioxide partial pressure (PaCO2) > 65 mmHg; poor perfusion, hemodynamic instability (i.e., mean blood pressure below gestational age) or both; needing volume or vasopressor support for 4 h or more; metabolic acidosis does not respond to treatment.

The exclusion criteria were as follows: congenital airway malformations, cleft lip and palate, Pierre-Robin syndrome, congenital diaphragmatic hernia, congenital lung dysplasia, tracheoesophageal fistula, and other life-threatening congenital malformations. Infants who failed to complete the treatment were excluded from the statistical data.

After informed consent was obtained, a total of 94 VLBWI were ultimately enrolled in the study, with 48 infants in the HHHFNC group and 46 infants in the NCPAP group through block randomization. Randomization was implemented by a random number generator and a special double-sealed envelope. When an infant met the admission criteria, the envelope was opened, and the treatment was immediately initiated.

**Figure 1:**
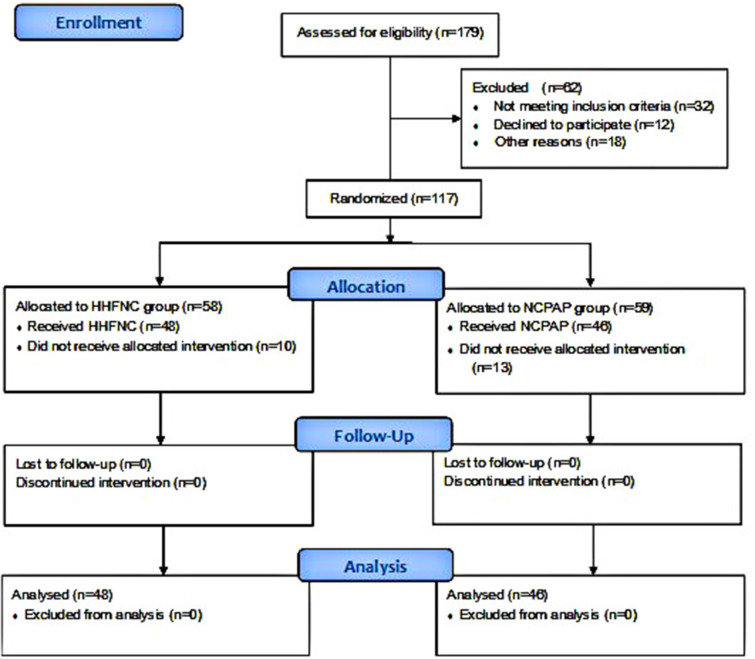
Flow chart of participants throughout the study.

All researchers were blinded to the randomized group assignment, but the co-researcher monitored the intervention procedure. A flow diagram of the study is shown in Figure 1.

## Materials and Methods

The criteria for the removal of invasive ventilation were as follows: HFOV mode: mean airway pressure (MAP) of 6–8 cmH_2_0, oxygen concentration (FiO_2_) ≤ 40%, and amplitude of 12–16; synchronized intermittent ventilation mode: MAP < 8 cmH_2_0, FiO_2_ ≤ 40%, ventilation frequency of 30 times/min; children have good spontaneous breathing; stable circulation; and less secretion.

Non-invasive assisted ventilation failure was indicated by the following: (1) PEEP > 8 cmH_2_O or FiO_2_ > 60% still cannot maintain percutaneous SaO_2_ ≥ 88%; (2) severe apnoea: >6 times within 24 h or >2 times of positive pressure ventilation after resuscitation; (3) the infant's breathing cycle could not be maintained or the infant was in shock; (4) severe metabolic acidosis or respiratory acidosis could not be corrected; (5) abdominal guarding and obvious abdominal distension (24-h increase in abdominal circumference greater than 1.5 cm) accompanied by one of a, b, c, d, and e: a. poor response, with blood sugar fluctuations; b. gastrointestinal bleeding; c. metabolic acidosis (BE < −10 mmol/L); d. body temperature instability; and e. significant increase in apnoea and bradycardia. In any of the above cases, tracheal intubation was performed again, and synchronized intermittent ventilation was performed. After re-intubation, the extubation was still changed to the original non-invasive ventilation mode.

The criteria for removal of non-invasive ventilation were as follows: chest X-ray and clinical improvement of the child and regular percutaneous SaO_2_ and blood gas analysis. The ventilator parameters of the HHHFNC group were reduced to flow < 2 L/min and FiO_2_ < 25%; the ventilator parameters of the NCPAP group were reduced to flow rate PEEP < 4 cmH_2_O and FiO_2_ < 25%.

HHHFNC group: powered by a Bird Air Oxygen Mixer (BIRD, USA), connected to an Optiflow™ Nasal Catheter Oxygen System (Fisher & Paykel Medical, New Zealand), including an MR850 warming humidifier, an RT329 high-performance closed breathing tube, and a short nasal plug catheter; a nasal plug of the right size was chosen. Initial parameters: FiO_2_ 30–40%, flow 4–6 L/min, heated, humidified inhalation gas at 37°C.

NCPAP group: powered by an Infant Flow System (EME Company, the United Kingdom). Initial adjustment parameters: flow 4–8 L/min, PEEP 5–7 cmH_2_O, FiO_2_ 40%, when the parameter is reduced to PEEP < 4 cmH_2_O, FiO_2_ < 0.25 can be withdrawn. The ventilator parameters were adjusted based on the improvement of clinical symptoms and blood gas results to maintain PaO_2_ 60–80 mmHg, PaCO_2_ 40–50 mmHg, and TcSaO_2_ 88–92%.

### Outcome Measures

Demographic and clinical characteristics were recorded, including age (weeks), birth weight (g), sex, Apgar scores, albumin (g/L), initial feeding time (d), mother's age (years), delivery, births, and antenatal use of corticosteroids.

Primary outcome measures included the reintubation rate within 7 days after initial extubation, total ventilation time, non-invasive ventilation time, and total oxygen inhalation time.

Secondary outcome measures included the time to reach full enteral feeding (day), average weight gain rate (g/day), days of hospitalization (day), and cost of hospitalization (thousand dollars).

Complications included intracerebral hemorrhage, retinopathy of prematurity, patent ductus arteriosus, bronchopulmonary dysplasia, necrotizing enterocolitis, and nasal injury.

### Descriptive Statistics

Data processing was done by statisticians who were not involved in the research design and implementation. The means ± standard deviations (SDs) for numerical variables and the percentages of different categories were obtained. Student's *t*-test or the Mann-Whitney *U*-test with a Chi-square test or Fisher's exact test was selected as appropriate. Tests of normality and homogeneity of variances were performed before comparisons between the measurement data groups. All data were analyzed using SPSS version 25.0 (SPSS, Chicago, IL, USA). A *P* < 0.05 was considered statistically significant.

#### Data Safety Monitoring Board

The board will have the following members:

Dr. Chuan Nie, Professor of Pediatrics; Neonatal Department, Guangdong Women and Children Hospital, Guangzhou.
Dr. Xiu Zhen Ye, Professor of Pediatrics; Neonatal Department, Guangdong Women and Children Hospital, Guangzhou.
Dr. Chun Shuai, Professor of Pediatrics; Neonatal Department, Guangdong Women and Children Hospital, Guangzhou.

They were arranged to conduct a simple mid-term evaluation. And they found that the trial was safe at midterm and agreed to continue.

## Results

### Demographic and Clinical Characteristics

None of the infants in the two study groups were lost to follow-up. As shown in Table 1, the demographics of infants were not statistically different between the two groups. Among the 94 infants, the majority of infants were males (59/94, 62.77%), and the mean age of all infants was 27.3 ± 3.10 weeks (range 25.1–32.0 weeks).

**Table 1:** Demographic and clinical characteristics of infants in the two study groups.

**Demographic**		**Groups [*****N*** **(%)]**			
**Variables**		**HHHFNC group [*N* = 48]**	**NCPAP group [*N* = 46]**	**In total [*N* = 94]**	***P*-value**
**DEMOGRAPHIC**					
Gestational age (weeks)	Mean ± SD	27.2 ± 2.8	27.5 ± 3.2	27.3 ± 3.1	0.724^b^
	Range (Mix–Max)	25.2–32.0	25.1–31.5	25.1–32.0	
Birth weight (g)	Mean ± SD	827 ± 23.0	794 ± 31.0	814 ± 27.0	0.218^b^
	Range (Mix–Max)	740–990	720–970	720–990	
Sex	Male	30 (62.5)	29 (63.04)	59 (62.77)	0.957^a^
	Female	18 (37.5)	17 (36.96)	35 (37.23)	
Apgar scores		5.2 ± 0.6	5.4 ± 0.4	5.3 ± 0.8	0.936^b^
Albumin	(g/L)	30.9 ± 2.9	31.4 ± 3.7	31.1 ± 2.8	0.342^b^
Initial feeding time	Day	3.25 ± 1.22	3.64 ± 1.35	3.44 ± 1.31	0.054^b^
**Variables**	**Yes/no**	***N*** **(%)**	***N*** **(%)**	***N*** **(%)**	***P*****-value**
**CLINICAL CHARACTERISTICS**					
Mother's age (years)		32.7 ± 5.1	33.1 ± 4.8	32.9 ± 5.0	0.517^b^
Delivery	Spontaneous delivery	14 (29.17)	13 (28.26)	27 (28.72)	0.923^a^
	C-section	34 (70.83)	33 (71.74)	67 (71.28)	
Births	Single	38 (79.17)	37 (80.43)	75 (79.79)	0.878^a^
	Multiple	10 (20.83)	9 (19.57)	19 (20.21)	
Small for gestational age	No	39 (81.25)	38 (82.61)	77 (81.91)	0.532^a^
	Yes	9 (18.75)	8 (17.39)	17 (18.09)	
Antenatal use of corticosteroids	No	10 (20.83)	10 (21.74)	20 (21.28)	0.544^a^
	Yes	38 (79.17)	36 (78.26)	74 (78.72)	
Extubation age (weeks)	Mean ± SD	27.8 ± 2.2	28.2 ± 2.6	31.8 ± 4.3	0.422
	Range (Mix–Max)	25.5–33.0	25.4–32.5	25.4–33.0	

*SD, standard deviation; HHHFNC, Heated, Humidified High Flow Nasal Cannula; NCPAP, Nasal Continuous Positive Airway Pressure*.

a *Chi-squared test or Fisher exact test*.

b *Student's t-test or Mann-Whitney U-test*.

### Primary Outcomes

Compared with the NCPAP group, the total oxygen consumption time in the HHHFNC group was significantly reduced, and the difference was statistically significant (*P* < 0.05).

There were no significant differences in total ventilation time, non-invasive ventilation time, and reintubation rate within 72 h (*P* > 0.05, see Table 2).

**Table 2:** Comparison of ventilation related factors between the HHHFNC group and the NCPAP group.

**Variables**		**Groups**			
**Number of patients**		**HHHFNC group [*N* = 48]**	**NCPAP group [*N* = 46]**	**Statistics test**	
		**Mean ± SD**	**Mean ± SD**	***U*-value**	***P*-value**
Re-intubation rate within 72 h	Yes	11 (22.91)	11 (23.91)	0.013	0.909
	No	37 (77.09)	35 (76.09)		
Total ventilation time	Day	19.4 (11.2–24.7)	17.9 (8.3–23.6)	0.102	0.645^a^
Non-invasive ventilation time	Day	12.7 (6.4–19.2)	10.8 (4.6–18.4)	0.518	0.337^a^
Total oxygen time	Day	29.7 (24.9–41.6)	32.1 (25.2–44.0)	3.074	0.030^a^

*SD, standard deviation; HHHFNC, Heated, Humidified High Flow Nasal Cannula; NCPAP, Nasal Continuous Positive Airway Pressure*.

a *Student's t-test or Mann-Whitney U-test*.

### Secondary Outcomes

Compared with the NCPAP group, the time to reach full enteral feeding (31.24 ± 11.30 vs. 34.21 ± 14.09 days) in the HHHFNC group was significantly earlier (*P* < 0.05). The average weight gain rate (16.07 ± 3.10 vs. 13.74 ± 4.21; g/day) was increased, the days of hospitalization (73.45 ± 18.84 vs. 79.24 ± 19.75) (days) were fewer, and the cost of hospitalization (16.04 ± 3.64 vs.18.79 ± 4.13; thousand dollars) was reduced (see Table 3).

**Table 3:** Related factors between the HHHFNC group and the NCPAP group.

**Variables**		**Groups**			
**Number of cases (%)**		**HHHFNC group [*N* = 48]**	**NCPAP group [*N* = 46]**	**In total [*N* = 94]**	***P*-value**
Total intestinal feeding time	Day	31.24 ± 11.35	34.21 ± 14.09	3.591	0.019^a^
Average weight gain rate	g/day	16.07 ± 3.10	13.74 ± 4.21	−2.804	0.040^a^
Days of hospitalization	Day	73.45 ± 18.84	79.24 ± 19.75	3.047	0.036^a^
Costs of hospitalization	Thousand dollars	16.04 ± 3.64	18.79 ± 4.13	2.748	0.001^a^

*SD, standard deviation; HHHFNC, Heated, Humidified High Flow Nasal Cannula; NCPAP, Nasal Continuous Positive Airway Pressure*.

a *Student's t-test or Mann-Whitney U-test*.

### Complications

The incidence rates of nasal injury (6.25 vs. 36.96%) and NEC (10.42 vs. 28.26%) in the HHHFNC group were significantly lower than those in the NCPAP group. The difference between the two groups was statistically significant (*P* < 0.05). There were no significant differences in the incidence rates of BPD, ROP, intracranial hemorrhage, PVL, and PDA between the two groups (*P* > 0.05, see Table 4).

**Table 4:** Comparison of complications in infants in the HHHFNC group and the NCPAP group.

**Variables**	**Groups**						
	**HHHFNC group**	**NCPAP group**	**Statistics test**				
**Number of cases *N* (%)**	***N* = 48**	***N* = 46**	**χ2**	**OR**	**95%CI**	**Regression coefficients**	***P*-value**
Intracerebral hemorrhage	7 (14.58)	7 (15.21)	0.007	0.951	0.331–2.961	−0.050	0.931
	41 (85.42)	39 (84.79)					
Retinopathy of prematurity	17 (35.42)	18 (39.13)	0.139	0.853	0.369–1.970	−0.159	0.710
	31 (64.58)	28 (60.87)					
Patent ductus arteriosus	16 (33.33)	16 (34.78)	0.022	0.938	0.399–2.201	−0.065	0.882
	32 (66.67)	30 (65.22)					
Bronchopulmonary dysplasia	16 (33.33)	15 (32.61)	0.006	1.033	0.437–2.443	0.033	0.904
	32 (66.67)	31 (67.39)					
Necrotizing enterocolitis	5 (10.42)	13 (28.26)	4.505	0.295	0.096–0.911	−1.220	0.034
	43 (89.58)	33 (71.74)					
Nasal injury	3 (6.25)	17 (36.96)	10.529	0.114	0.031–0.423	−2.174	0.001
	45 (93.75)	29 (63.04)					

*SD, standard deviation; HHHFNC, Heated, Humidified High Flow Nasal Cannula; NCPAP, Nasal Continuous Positive Airway Pressure; CI, confidence interval*.

## Discussion

NCPAP is the earliest non-invasive respiratory support for postpartum extubation (10). It can keep the airway in an expanded state, prevent alveolar collapse and improve the ventilatory blood flow ratio. Distributing an accurate pressure for variable flow through CPAP involves a tightly sealed nasal interface. However, if it is too tight, the possibility of skin rupture and mucosal damage is greater. In contrast, the key mechanism of HHHFNC is to wash out the nasopharyngeal dead space with humidified and warm gas (11); for that reason, a gap between the nasal cannula and nares is required to wash out the gas. Hence, the direct pressure effect between the proper size of the cannula of HHHFNC and the nares is much weaker than that of CPAP nasal interfaces, resulting in less nasal trauma. In the current study, as shown in Table 4, the incidence rates of nasal injury (6.25 vs. 36.96%) and NEC (10.42 vs. 28.26%) in the HHHFNC group were significantly lower than those in the NCPAP group. The difference between the two groups was statistically significant (*P* < 0.05).

A meta-analysis of randomized controlled trials published in 2019 showed that for respiratory support after extubation, NCPAP was associated with a lower likelihood of treatment failure than high-flow nasal cannula (HFNC) (relative risk 1.23, 95% confidence interval 1.01–1.50). The incidence rates of nasal trauma and pneumothorax in the HFNC group were significantly lower than those in the NCPAP group (*P* < 0.0001 and *P* = 0.03) (12).

Due to the pressure produced by the cumbersome and heavy dressing of the head and face with the NCPAP, it is easy to cause the nasal compression, the nasal skin to be damaged, the nostrils to expand and deform, and the nasal mucosa to develop oedema, congestion, and other damage in infants. Nasal congestion can irritate the nostrils and increase the secretions in the nasal cavity, increasing the risk of nasal and systemic infections, especially for ELBWI. In another systematic review and meta-analysis article published in 2020, Junior et al. also showed non-inferiority in terms of therapeutic failure of HFNC in relation to NCPAP after extubation of preterm newborns. In addition, nasal trauma was significantly lower in patients submitted to the HFNC compared to those using NCPAP (*P* < 0.0001) (13).

Compared with NCPAP, HHHFNC is a simple device that directly places the nasal cannula for the right side of the nose into the nasal cavity and gets rid of the external force on the head and face, thus avoiding head deformation and nasal injury (Supplementary Figures 1–3) (14). Similarly, these results are supported by a meta-analysis that revealed that nasal mucosa injury scores were significantly lower for HHHFNC compared to other methods of non-invasive ventilation (15). Similarly, it was also confirmed that the incidence of nasal injury in the HHHFNC group was significantly lower than that in the NCPAP group (*P* < 0.05), indicating that HHHFNC can effectively prevent nasal injury.

In addition to the low weight of the HHHFNC apparatus, HHHFNC has a relatively high oxygen humidification rate. If there is inadequate warming and humidification, a large amount of high-flow dry and cold air will enter the nasal cavity of the child, causing damage and bleeding of the nasal mucosa, which will greatly increase the chance of infection. In our study, the hollow oxygen mixed gas passed through a Fisher & Paykel MR850 heating humidifier, and the gas delivered through the closed breathing circuit was supplemented with molecular water vapor with a temperature of ~37°C and a relative humidity of nearly 100%. As shown in Table 2, compared with the NCPAP group, the total oxygen consumption time in the HHHFNC group was significantly reduced, and the difference was statistically significant (*P* < 0.05).

Saslow et al. (16) found that the improvements in respiratory work and lung compliance in preterm infants were comparable to the NCPAP 6 cmH_2_O when the HHHFNC flow reached 5 L/min. Moreover, some studies (17, 18) have also shown that the HHHFNC apparatus is lighter than NCPAP devices, but the pressure generated by breathing is close to the pressure generated by NCPAP. This makes it possible for HHHFNC to replace NCPAP as non-invasive respiratory support after extubation in ELBWI. Recent studies have indicated that with a flow rate of 4–6 L/min and a suitable nasal cannula size, a diameter ~50–80% of that of the infants' nares would be safe for preterm infants (6, 19, 20). A meta-analysis also presented no differences in pulmonary air leakage or mortality between HHHFNC and other forms of non-invasive respiratory support (15). Osman et al. (21) scored pain in infants with HHHFNC and NCPAP and found that infants in the HHHFNC group had significantly less pain and improved tolerance.

This study confirmed that the use of HHHFNC for assisted ventilation after extubation was significantly shorter than that of NCPAP, and the number of infants who were reintubated was significantly less than that of the NCPAP group. This is consistent with the findings of Woodhead et al. (22) that HHHFNC can reduce respiratory work and reduce the rate of reintubation.

Abdominal distension and NEC are also important factors that cause non-invasive ventilation failure in preterm infants and that require re-intubation. This study confirmed that the incidence rates of NEC in the NCPAP group were significantly higher than those in the HHHFNC group, and the differences were statistically significant (*P* < 0.05), which resulted in a significantly longer time to reach full enteral feeding in the NCPAP group than in the HHHFNC group (*P* < 0.05). ELBWI should start drinking breast milk as soon as possible, and the time to reach full enteral feeding can promote the secretion of gastrointestinal hormones and intestinal movement, which are beneficial for the balance of enteral nutrition and protein/energy (23). Therefore, HHHFNC is more conducive to healthy infant weight gain than NCPAP, which can improve the long-term quality of life of children.

This study also confirmed that HHHFNC reduced the length of the hospital stay and significantly reduced hospitalization costs. These reductions were significantly smaller in the HHHFNC group than in the NCPAP group. The initial feeding time in the HHHFNC group was earlier than that in the NCPAP group. The daily weight gain rate was faster and the time to reach full enteral feeding was earlier in the HHHFNC group than in the NCPAP group. This study also indicated that there were no significant differences in the incidence of complications such as total ventilation and BPD, ROP, PDA, PVL, and intracranial hemorrhage (*P* > 0.05). Moreover, HHHFNC has a significantly lower unit price per hour than NCPAP, making it very beneficial for low- and middle-income families.

A possible limitation of this study is that HHHFNC cannot directly detect the actual pressure of the given flow parameters and whether the thickness of the nasal catheter used directly affects the clinical efficacy.

## Conclusion

In summary, compared with the use of NCPAP, HHHFNC can significantly reduce the reintubation rate within 7 days, shorten the oxygen exposure time, and significantly reduce the incidence of complications such as nasal injury and NEC. HHHFNC did not increase the incidence of BPD, ROP, PDA, PVL, or intracranial hemorrhage in infants. Moreover, HHHFNC shortened the length of hospital stays for infants, greatly reduced hospitalization costs, and can greatly reduce the medical burden on low- and middle-income families. However, multi-center, large-sample randomized controlled clinical trials on the mechanism of action of HHHFNC are needed to further explore its safety and efficacy.

## Data Availability Statement

All datasets generated for this study are included in the article/Supplementary Material.

## Ethics Statement

The studies involving human participants were reviewed and approved by the institutional review board of the Guangdong Women and Children Hospital (Guangzhou, China). Written informed consent to participate in this study was provided by the participants' legal guardian/next of kin. Written informed consent was obtained from the individual(s), and minor(s)' legal guardian/next of kin, for the publication of any potentially identifiable images or data included in this article.

## Author Contributions

JC and WG conceptualized the study, drafted the initial manuscript, and reviewed and revised the manuscript. YLin and MK collected data, carried out the initial analyses, and reviewed and revised the manuscript. LD and ZW processed the experimental data. XC and YLiu mainly discussed and edited the manuscript. JY and YC designed the data collection instruments, coordinated and supervised data collection, and critically reviewed the manuscript. All authors approved the final manuscript as submitted and agree to be responsible for all aspects of the work.

## Conflict of Interest

The authors declare that the research was conducted in the absence of any commercial or financial relationships that could be construed as a potential conflict of interest.

## Abbreviations

ELBWI: Extremely low-birth-weight preterm infants
HHHFNC, Heated: humidified high-flow nasal cannula
NCPAP: Nasal continuous positive airway pressure
IVH: Intraventricular hemorrhage
ROP: Retinopathy of prematurity
PDA: Patent ductus arteriosus
BPD: Bronchopulmonary dysplasia
NEC: Necrotizing enterocolitis
CI: Confidence interval.
