# Supplementary material for: The Comparison of HHHFNC and NCPAP in Extremely Low-Birth-Weight Preterm Infants After Extubation: A Single-Center Randomized Controlled Trial
Source: Front Pediatr. 2020 Jun 26;8:250. doi: 10.3389/fped.2020.00250 (PMC7332541; doi:10.3389/fped.2020.00250)

**Image 1. Nasal injury of an extremely low birth weight preterm infant**


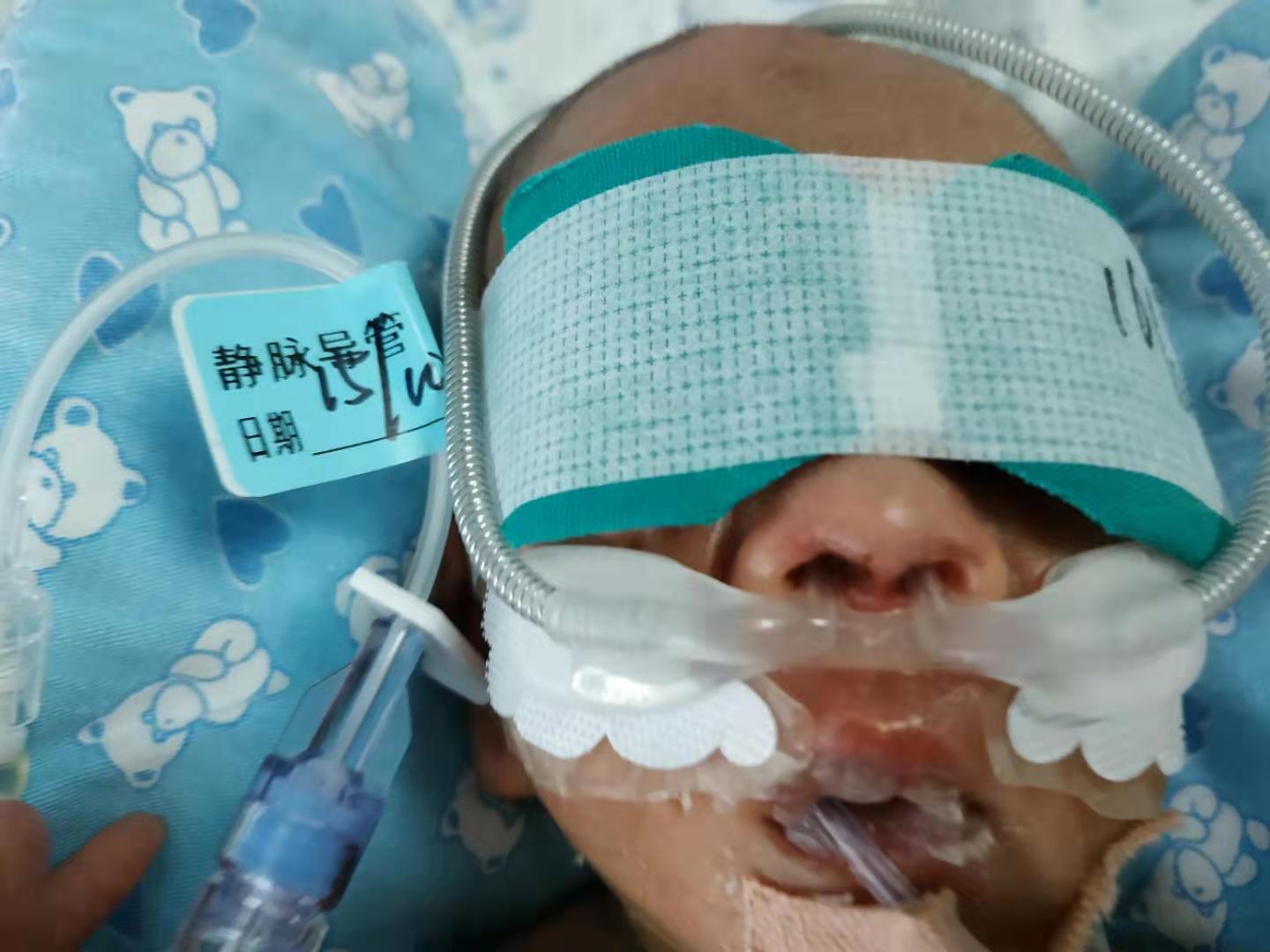


**Notes: The use of CPAP initially caused nasal injury, now using HHFNC.**

**Image 2. After the nasal injury has improved of the infant**


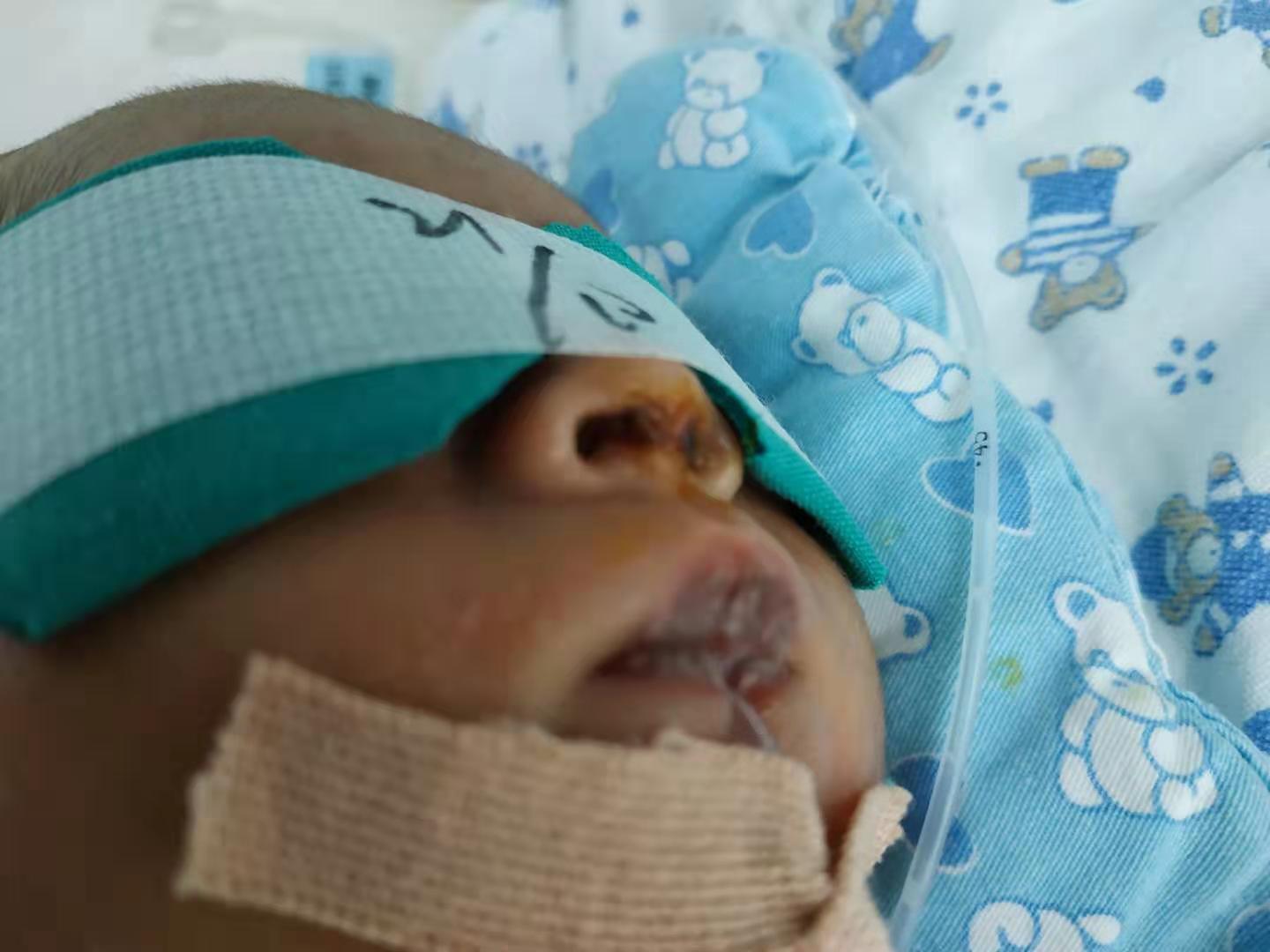


**Image 3. The infant's nasal injury is fully restored (before discharge)**


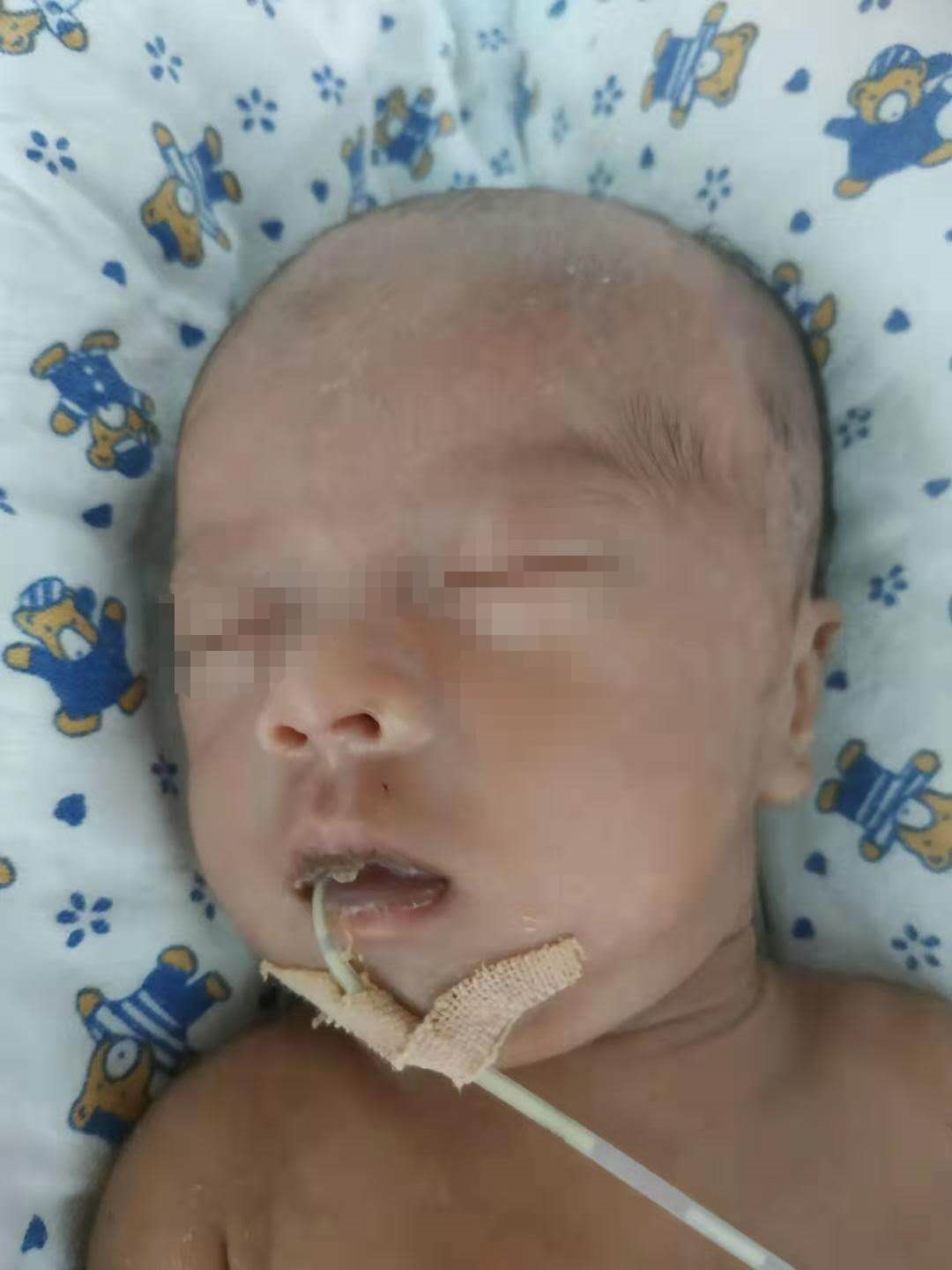

Supplement: Supplementary file 1 [file Data_Sheet_1.docx]
